# Influence of Temperature, Humidity and Load Coupling on Mechanical Properties of Adhesive Joints and Establishment of Creep Model

**DOI:** 10.3390/polym15020339

**Published:** 2023-01-09

**Authors:** Wei Tan, Zhaofeng Zhou, Jingxin Na, Wenlong Mu

**Affiliations:** 1 Department of Mechanical Engineering, Hangzhou City University, Hangzhou 310015, China; 2 State Key Laboratory of Automotive Simulation and Control, Jilin University, Changchun 130031, China; 3 College of Mechanical & Electrical Engineering, Henan Agricultural University, Zhengzhou 450002, China

**Keywords:** polyurethane adhesive, shear joint, creep model, property degradation, failure mechanism

## Abstract

To study the creep and property degradation behavior of adhesive joints under the coupling action of temperature, humidity and load, polyurethane shear joints were prepared and tested. Different static loads were applied to joints at high temperature (80 °C) and high temperature and humidity (80 °C/95% RH) to test and analyze the creep deformation, and a suitable creep model was established. At the same time, the performance degradation test of the joints under the effect of multifactor coupling was carried out to obtain the variation law of the failure load, and the failure mechanism was discussed based on the failure section. The research shows that the creep strain of the joint at high temperature and humidity was significantly larger than that at high temperature, and the failure fracture time was shorter, in which water molecules played a role of softening and hydrolysis. The viscoelastic multi-integral creep model was used to analyze and predict the creep behavior of the joints. It was found that the creep model could better describe the creep behavior of the joints under uniaxial constant loading. Under the coupling effect of temperature, humidity and load, the failure load decreased with time, and with the increase in static load, the decline range and rate of failure load increased. It was found that the mechanical properties in the high temperature and humidity environment decreased significantly more than those in the high temperature environment. When a static load was applied during creep, cracks easily occurred inside the adhesive layer, and water molecules easily diffused inside the cracks, which increased the decay rate of the mechanical properties. This study provides good theoretical significance and engineering value for the application of polyurethane adhesion structures in rail vehicles.

## 1. Introduction

With the development of railway technology, the requirements for lightweight rail vehicles are increasingly high, especially with the emergence of new materials and technologies. A large number of light metal materials and composite materials have replaced traditional metal materials, which is an effective means to achieve light weight. The mixed application of various materials is the inevitable trend of lightweight technology development [1]. The traditional connection methods had difficulty meeting the demand, such as welding and riveting, etc. [2,3]. However, adhesion has the advantages of fatigue resistance, good sealing and vibration reduction [4] and can also resolve the electrochemical corrosion problem in the connection of dissimilar materials [5]. In recent years, adhesion technology has increasingly been used to replace the traditional bonding process for the connection of rail train materials, which plays a very important role in the body structure connection.

Surface pretreatment is one of the most important links in adhesive bonding. Rudawska et al. [6] evaluated the effect of surface treatment on the bonding performance of steel plate surface and joint strength, and found that the surface of degreased steel plate has better wettability with both polar and apolar fluids. Gonzalez-Canche et al. [7] characterized the surface treatment through wettability, morphology and chemical analysis, and found that the treatment of metal layers by a sodium hydroxide and nitric acid pickling process improved the degree of adhesion and tensile strength of fiber metal laminates. Miturska-Barańska et al. [8] measured the influence of the sandblasting process as a surface preparation method on the strength of single-lap adhesive joint of aerospace aluminum alloy sheets, and evaluated the influence of surface preparation method on the surface roughness and contact angle to determine the surface free energy.

For a polymer compound, the temperature and humidity in the environment are the main factors causing material aging [9]. The adhesives are polymers with hygroscopicity. Under hot and humid conditions, water molecules are more likely to enter the colloid and accelerate aging. Meanwhile, hygroscopic expansion will also produce internal stress, causing changes in mechanical properties [10]. Water absorption of the adhesive will cause plasticization and expansion under the coupling effect of temperature and humidity. In addition to the increase in strain, the degradation of water will significantly reduce the strength, stiffness, fracture toughness and other properties of the adhesive [11]. Rudawska et al. [12] studied the influence of aqueous solutions with different FeSO_4_ contents on the mechanical properties of adhesive joints, analyzed the compression modulus, strength and strain, and found that excess FeSO_4_ content in water had a negative impact on the mechanical properties of adhesives. Heshmati [13] and VIANA [14] quantitatively evaluated the aging behavior of the adhesives and joints in a hygrothermal environment, and analyzed the fracture mechanism. Bellini et al. [15] believed that humidity and temperature were the main factors affecting the durability of joints. Three aging environments were selected (thermal cycles from −28 °C to 85 °C in air, distilled water and salt water) to study the influence of hydrothermal ageing test on the mechanical properties of CFRP joints.

Adhesive structure as a load-bearing structure will bear static load for a long time. The adhesive will experience creep deformation, which affects the mechanical properties of the adhesive structure [16], making it fail when the force is less than the failure load— seriously affecting the safety of the train [17]. Agarwal et al. [18,19] studied the effect of coupling aging on the properties of CFRP/steel joints through temperature cycling (10–40 °C/10–50 °C), static load (30% and 50% static failure load), and water immersion. It was found that single temperature, load, and moisture had little effect on the joint properties, but the strength decreased by approximately 15% under the combined action of moisture and heat and by approximately 47% under the coupling action of temperature, load, and moisture. Han et al. [20] analyzed the strength degradation of joints under multifactor coupling conditions and found that water significantly reduced the fracture load, while the intervention of continuous load further expanded its influence.

Domestic and foreign scholars have focused on creep experimental research of adhesive structures and the use of different creep models to describe the creep properties of adhesive structures. However, there are relatively few studies on the effects of humid and thermal environments on the creep behavior of polyurethane adhesive structures, especially the comparative analysis of the effects of creep properties under different environments and stress states [21,22]. Therefore, it is very important to establish a suitable creep model to describe the creep behavior of polyurethane adhesives under different environments and stress levels. The nonlinear viscoelastic creep models of materials mainly include single integral, multiple integral, power rate model and differential integral models. Among them, the Onaran model with multiple integrals, Lianis model with single integrals, and Findley model with power rate are widely used [23]. Galvez et al. [24] studied the ability of adhesives to cope with temperature (72 °C) and humidity (82%) and temperature variations (from 26 °C to 72 °C) through durability and thermal aging tests, and analyzed the changes generated during bonding by differential scanning calorimetry and infrared spectroscopy. Dean [25] studied the creep behavior of bonded joints in hygrothermal environments using rubber toughening adhesives. It was found that the creep rate of joints in humid and hot environments was significantly higher than that in dry environments, and the exponential function based on relaxation time and short-term creep compliance could be used to simulate the creep compliance curves.

In conclusion, it is of great engineering application value to research the aging and creep characteristics of the adhesive joints under different temperature and moisture environments. The shear joints are selected as the research object, and static loads of different load levels are applied to the joints under different temperature and moisture environments. This paper discusses the creep phenomenon and property degradation behavior, analyzes the creep deformation and failure mechanism, establishes a suitable creep model, and discusses the change rule of mechanical properties.

## 2. Experimental Study

### 2.1. Material

A 6005A aluminum alloy was selected as the bonding substrate. The main component of 6005A aluminum alloy is Al–Mg–Si, which is a kind of medium-strength aluminum alloy with good formability, corrosion resistance and weldability, which is the main material to be widely used by rail trains. The adhesive was Sikaflex^®^-265, a single-component polyurethane adhesive which can be exposed to atmospheric humidity to complete self-curing. The T_g_ of the Sikaflex^®^-265 is −66 °C. The adhesive has excellent fatigue durability, high ductility, and impact resistance, which has a wide range of applications in rail trains, buses, and trucks. The basic technical parameters of 6005A aluminum alloy and Sikaflex^®^-265 adhesive (data provided by the supplier) are shown in Table 1. The chemical composition of the 6005A aluminum alloy is shown in Table 2.

### 2.2. Joint Design and Manufacture

**Figure 1:**
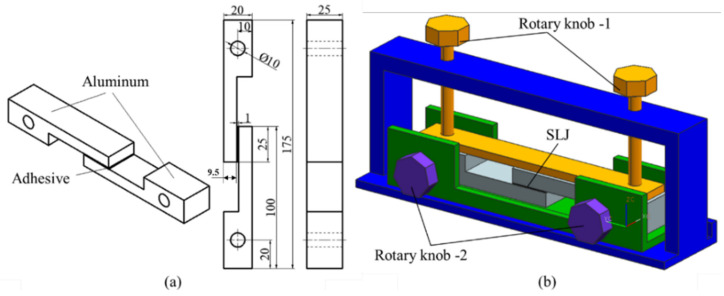
(**a**) Geometric dimension of the joint (unit: mm), (**b**) Schematic diagrams of the fixture.

With reference to the standard of <<Adhesives-Determination of tensile lap-shear strength of rigid-to-rigid bonded assemblies>> (GB/T 7124-2008), single lap joints can be selected for testing. However, when the matrix stiffness of the single lap joint is insufficient, the adhesive substrate will deform, and the actual internal stress direction will change, resulting in the adhesive not being subject to pure shear force. Therefore, considering the quasi-static and creep test conditions of joints, thick aluminum alloy was selected as the bonding substrate to prepare the shear joint. The joint size is shown in Figure 1a. The overall dimension was 175×25×20 mm^3^, the bonding area was 25×25 mm^2^ and the bondline thickness was 1 mm. Since the elastic modulus of the aluminum alloy was far greater than that of the adhesive, if the applied load was small during the creep test, it could be considered that the deformation of the substrate was very small, and the change in displacement mainly came from the adhesive deformation.

The samples were prepared in a dustless environment (temperature 25 ± 5 °C and relative humidity 50 ± 5%). To avoid bonding failure due to improper surface treatment, 80 mesh sandpaper was used to cross-polish the 6005A aluminum alloy along the diagonal direction of the bonding surface to increase the surface roughness. Then, Sika Remover 208, Sika Aktivator and Sika Primer-206G + P were applied to the adhesive surface [26]. Afterwards, adhesive was applied to the surface of the bonding substrate, and the shear joint was prepared through the fixture, as shown in Figure 1b. After 4 weeks of joint curing, the residual adhesive was cleaned.

### 2.3. Mechanical Properties Test

**Figure 2:**
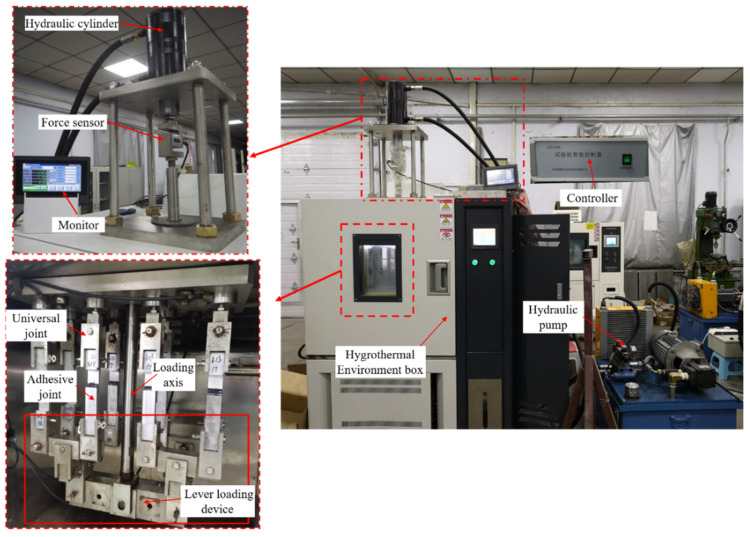
Environment and load coupling test device.

Different degrees of static loads were applied to joints under different environments. Quasi-static tensile tests were carried out on the joints with different periods of action to obtain the variation rules of mechanical properties with time. To test the influence of temperature, a high-temperature environment (80 °C) was selected by reference to standard [27], which was represented by “HT”. To test the influence of humidity, a high-temperature and high-humidity environment (80 °C/95% RH) was selected, which was represented by “HTAH”. Referring to the fatigue life curves obtained from the test in article [28], static loads of 200 N, 400 N and 600 N were applied to the joints, and the stress calculated from the adhesive area were 0.32 MPa, 0.64 MPa and 0.96 MPa. The self-developed environment and load coupling test device used the lever principle to load the joints (as shown in Figure 2). The hydraulic cylinder loaded eight specimens simultaneously through the primary, secondary and tertiary levers in turn. The dimensions of the lever and the joint were precisely designed and processed. Even if there was a small size error for each bonded specimen, the lever could balance the size error by rotating around the middle fulcrum, and the same load could still be applied to eight specimens through multi-level levers.

Through pre-experiment, it was found that the joints were not easy to fracture when a 200 N load was applied, and the test duration was longer. To study the mechanical properties at 200 N loading, 384 h was selected as the test period. When the joint was loaded with 400 N and 600 N, it was found that the joint failed and fractured in less than 384 h. Under the same test conditions, the time for two joints to fracture was the longest test cycle. The test scheme is shown in Table 3. The joints were removed from the test device at the end of each test period, and a quasi-static tensile test (electronic universal testing machine, WDW series, Ke Xin Co., Changchun, China) was conducted after 4 h at room temperature. The mechanical property curves were obtained through the tensile rate of 5 mm/min, and three joints were tested repeatedly under the same experimental conditions and period. There were 120 samples of adhesive joints actually tested.

### 2.4. Creep Performance Test

**Figure 3:**
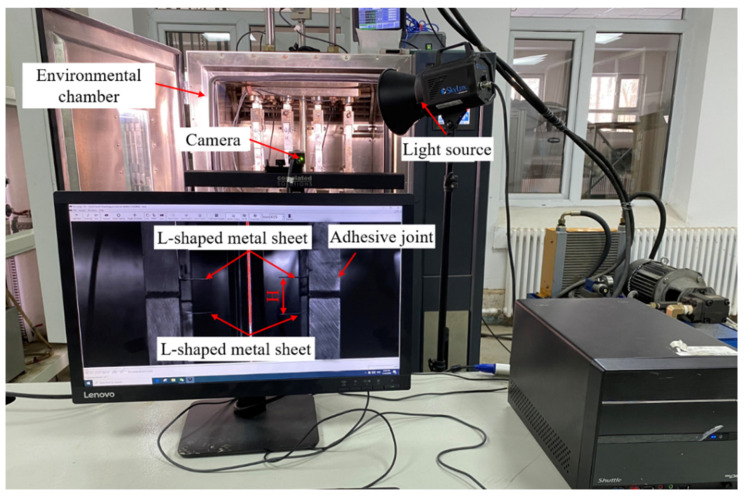
Creep test.

In combination with the VIC-3D (noncontact full field strain measurement system, Correlated Solutions Inc.), the joints were tested with the environment-load coupling test device, as shown in Figure 3. The test method was to fix the upper and lower parts of the joints with two L-shaped metal sheets, and test the change rule of the joint deformation with time. Pictures were taken every 5 s by VIC-3D, and the relative distance H between the L-shaped metal sheets was analyzed by the pixel method. The equipment was loaded at a speed of 100 N/s, and the joint could be loaded to the corresponding stress level within 10 s. Since the constant load applied during creep was far less than the elastic limit of the base material, the measured creep deformation mainly came from the adhesive.

## 3. Results and Analysis

### 3.1. Creep Performance Analysis

**Figure 4:**
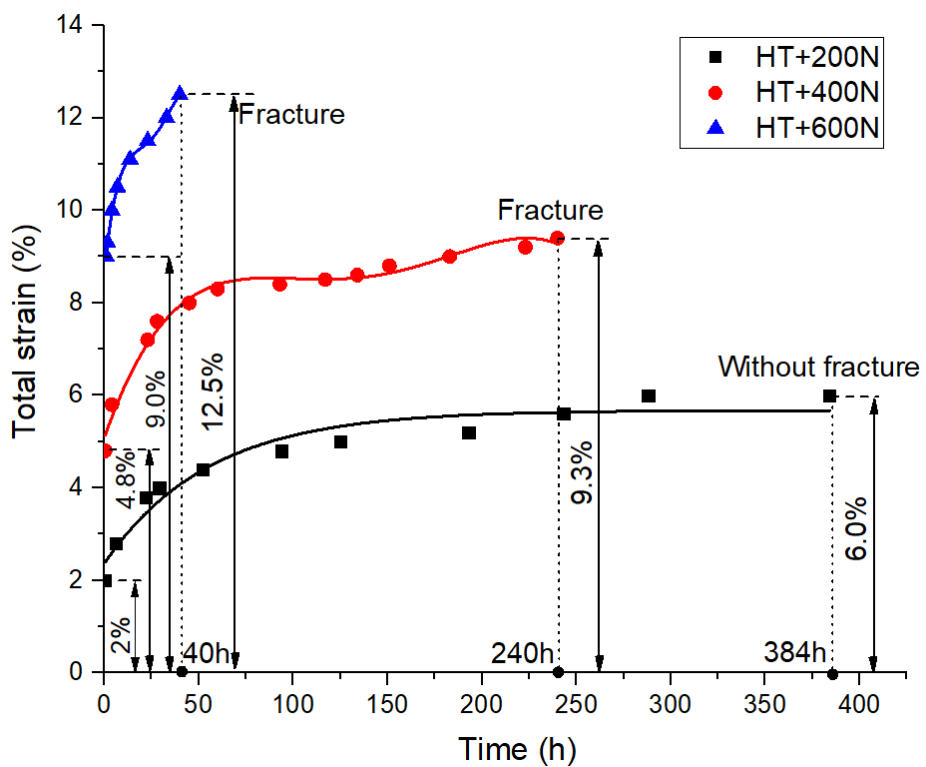
Creep curves in the HT environment.

**Figure 5:**
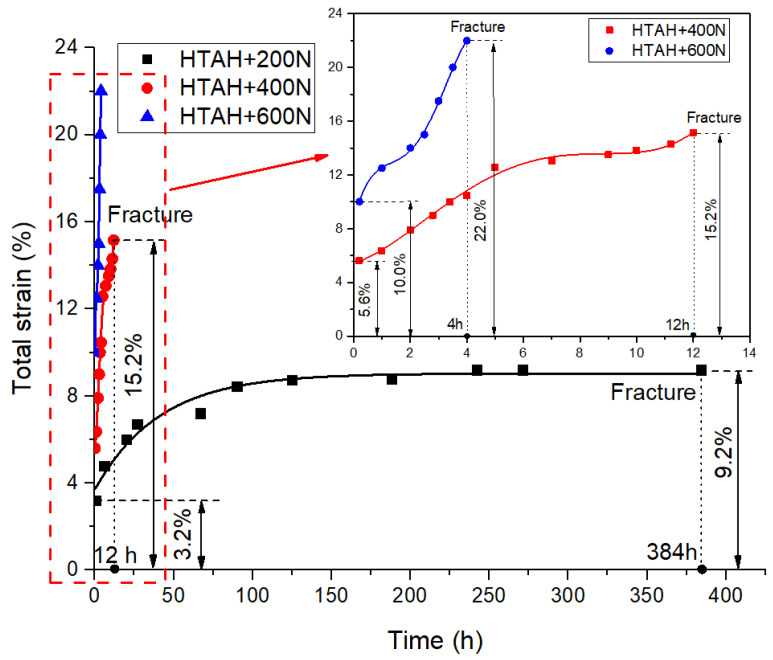
Creep curves in the HTAH environment.

The creep deformation of the joint was tested under 200 N, 400 N and 600 N static loads under different environments, and the variation of strains with time were obtained. The creep curves of joints under HT and HTAH environments are shown in Figure 4 and Figure 5, respectively. The creep experiment was divided into two stages. The first stage was to quickly load to the predetermined load, which is assumed to be the initial test time, and the second stage was to load with constant stress. It was found that the increase in load level increased the instantaneous strain and creep strain rate and resulted in greater creep strain and shorter fracture failure time. When 200 N, 400 N and 600 N were loaded respectively in the HT environment, the strain of the first stage was 2.0%, 4.8% and 9.0%, and the strain at fracture was 6.0%, 9.3% and 12.5%. When 200 N, 400 N and 600 N were loaded respectively in the HTAH environment, the strain of the first stage was 3.2%, 5.6% and 10.0%, and the strain at fracture was 9.2%, 12.5% and 22.0%. This result indicated that the strain variable and strain rate of the joint were larger under the HTAH environment, indicating that water played a softening and hydrolysis effect [29].

It was found that the joint strain increased slowly and then became stable after a loading of 200 N in the two environments, and the joint did not fracture after 384 h. When 400 N was loaded in the HT environment, the initial strain increased sharply, and the rate of increase decreased with time. When 600 N was loaded, it was observed that the joint exhibited obvious necking, resulting in the generation of cracks and holes in the adhesive layer, leading to an increase in the creep rate. However, when 400 N and 600 N were loaded in the HTAH environment, the creep rate was relatively large at the beginning, then the intermediate rate decreased, and finally increased gradually with time until fracture occurred. The results show that the influence of HTAH environment on joint creep was more obvious, and was related to the applied load. Because the plasticization of water played a dominant role at high humidity, it led to large creep strain.

### 3.2. Constitutive Theory of Creep Models

The viscoelastic multiple integral Onaran creep model was used to conduct theoretical analysis on the creep characteristics of joints [30], and the theoretical and measured values of the creep model were compared and analyzed. In the viscoelastic multi-integral creep model modified by Onaran et al. [30], its constitutive equation under uniaxial constant stress is as follows:

(1) $$\begin{array}{l} \varepsilon (t)=\int_{0}^{t}K_{1}(t-t_{1})\frac{\partial \sigma (t_{1})}{\partial t_{1}}dt_{1}+\int_{0}^{t}\int_{0}^{t}K_{2}(t-t_{1},t-t_{2})\frac{\partial \sigma (t_{1})}{\partial t_{1}}\frac{\partial \sigma (t_{2})}{\partial t_{2}}dt_{1}dt_{2}\\ +\int_{0}^{t}\int_{0}^{t}\int_{0}^{t}K_{2}(t-t_{1},t-t_{2},t-t_{3})\frac{\partial \sigma (t_{1})}{\partial t_{1}}\frac{\partial \sigma (t_{2})}{\partial t_{2}}\frac{\partial \sigma (t_{3})}{\partial t_{3}}dt_{1}dt_{2}dt_{3} \end{array}$$

where the first double integral term on the right of the equal sign is the linear response term, the second double integral term is the contribution to deformation generated by the interaction of every two increments, and so on, and $K_{1},K_{2},K_{3}$ are the creep flexibility.

The constitutive Equation (1) under uniaxial constant stress can be transformed into a uniaxial creep model as follows:

(2) $$\varepsilon (t)=K_{1}(t)\sigma +K_{2}(t)\sigma^{2}+K_{3}(t)\sigma^{3}$$

The expression of parameter $K_{1},K_{2},K_{3}$ is as follows:

(3) $$K_{1}(t)=\mu_{1}+\omega_{1}t^{n},K_{2}(t)=\mu_{2}+\omega_{2}t^{n},K_{3}(t)=\mu_{3}+\omega_{3}t^{n}$$

where $\mu_{1},\mu_{2},\mu_{3}$ are the constants related to the material, and $\omega_{1},\omega_{2},\omega_{3},n$ are the parameters related to the temperature, which are constant in a homothermic environment. According to the power-rate creep constitutive relation, the creep strain under constant stress satisfies the following equation:

(4) $$\varepsilon (t)=\varepsilon_{0}+\varepsilon_{t}t^{n}$$

where $\varepsilon_{0}$ is the initial strain, $\varepsilon_{t}$ is the strain related to stress, and *n* is a constant. Substituting Equation (3) into Equation (2) and comparing it with Equation (4), we can obtain:

(5) $$\varepsilon_{0}=\mu_{1}\sigma +\mu_{2}\sigma^{2}+\mu_{3}\sigma^{3}$$

(6) $$\varepsilon_{t}=\omega_{1}\sigma +\omega_{2}\sigma^{2}+\omega_{3}\sigma^{3}$$

The multiple integral creep model was used to analyze the creep characteristics of the joints. The core parameters of the constitutive equation were determined by comparing the experimental data, and the creep constitutive equation of the joints was obtained. The data measured through the creep experiment are converted into two co-ordinate systems to obtain $\varepsilon_{0},\varepsilon_{t}$ and n, which are substituted into Equations (5) and (6). Six core parameters are obtained through linear regression, and the multiple integral creep model shown in Equation (7) is obtained.

(7) $$\varepsilon (t)=(\mu_{1}+\omega_{1}t^{n})\sigma +(\mu_{2}+\omega_{2}t^{n})\sigma^{2}+(\mu_{3}+\omega_{3}t^{n})\sigma^{3}$$

### 3.3. Establishment of Creep Model

The multiple integral constitutive relation and Findley power rate relation are used to process the data in Figure 4 and Figure 5, and Equation (4) is used for term shifting and logarithm taking on both sides to obtain the first order linear function of ln(t), as shown in Equation (8):

(8) $$\ln\left[\varepsilon (t)-\varepsilon_{0}\right]=\ln\varepsilon_{t}+n\ln(t)$$

**Figure 6:**
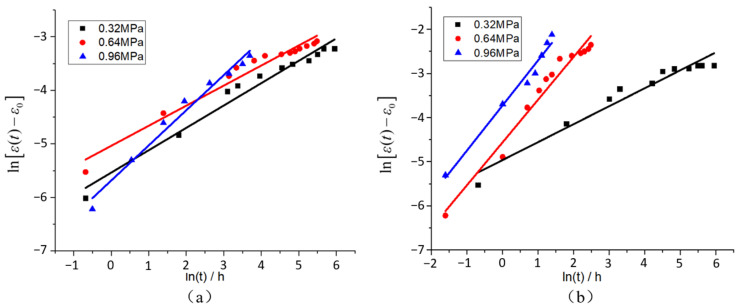
Creep strain fitting curve in double log coordinates: (**a**) HT, (**b**) HTAH.

Formula (8) shows that the slope of the fitting curve is *n* when $\ln\left[\varepsilon (t)-\varepsilon_{0}\right]$ is plotted against ln(t). When ln(t)=0, that is, t=1, $\varepsilon_{t}=\varepsilon (t)-\varepsilon_{0}$,$\varepsilon_{t}$ is the corresponding intercept. The constant-load creep curves of the joints under HT and HTAH conditions are processed, and the creep strain fitting curves of the joints under double logarithmic coordinates are obtained, as shown in Figure 6. By analyzing the fitting curve, *n*, $\varepsilon_{t}$ and $\varepsilon_{0}$ are obtained, as shown in Table 4.

The $\varepsilon_{t}$ values in Table 4 are substituted into Equation (6), and the corresponding values are obtained by linear regression, as shown in Table 5. Since the test is conducted under a constant temperature environment, the corresponding $\omega_{1},\omega_{2},\omega_{3}$ values are constant. Then, the initial strain $\varepsilon_{0}$ value in the table is substituted into Equation (5), and the corresponding $\mu_{1},\mu_{2},\mu_{3}$ values are obtained through linear regression calculation, as shown in Table 6.

By substituting the calculated values of n, ω and μ into Equation (7), the creep constitutive equations of joints can be obtained. Equations (9) and (10) are the creep constitutive equations of joints under HT and HTAH, respectively.

(9) $$\varepsilon \left(t\right)=(0.056+0.0105\cdot t^{n})\sigma +(0.0095+0.013\cdot t^{n})\sigma^{2}+(0.0056-0.021\cdot t^{n})\sigma^{3}$$

(10) $$\varepsilon \left(t\right)=(0.142+0.039\cdot t^{n})\sigma +(-0.177-0.0737\cdot t^{n})\sigma^{2}+(0.144+0.0615\cdot t^{n})\sigma^{3}$$

**Figure 7:**
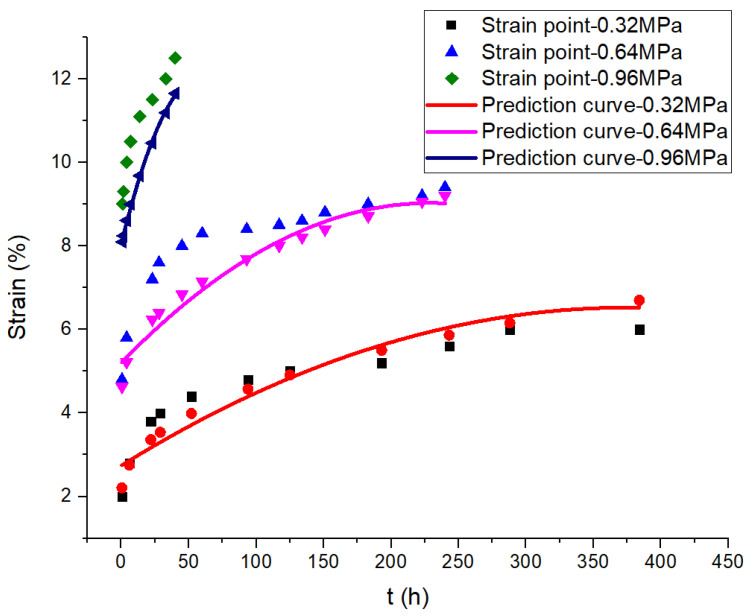
Comparison of strain points and predicted curves of creep in the HT environment.

**Figure 8:**
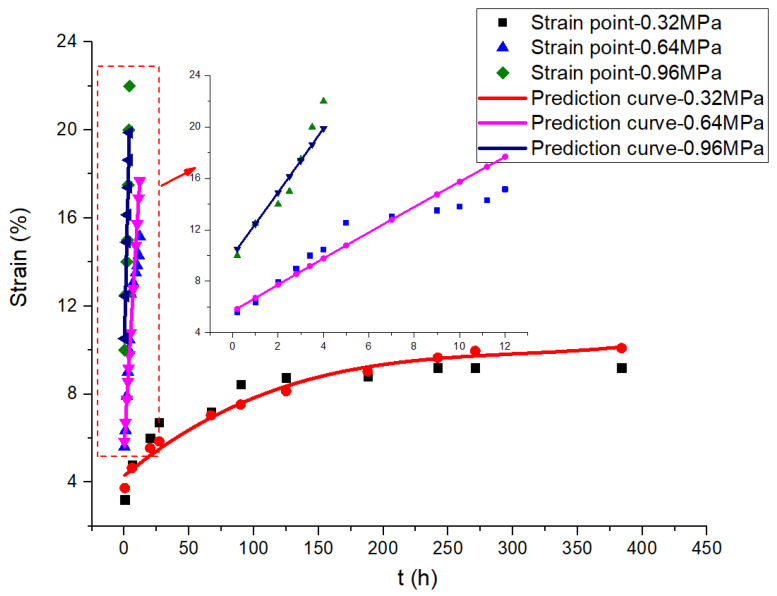
Comparison of strain points and predicted curves of creep in the HTAH environment.

To compare and analyze the theoretical value and the measured value of the creep curves of the joints, creep curves of 0.32 MPa, 0.64 MPa and 0.96 MPa under HT and HTAH environments are drawn for verification. The theoretical values of the joint strain are calculated according to Equations (9)–(10), and the comparison results with the measured values are shown in Figure 7 and Figure 8. It is found that the theoretical values of the strain of the joints coincide well with the measured value. In conclusion, the viscoelastic multi-integral Onaran constitutive model is used to analyze the creep characteristics of polyurethane shear joints, which can better describe the creep behavior of joints under uniaxial constant shear loads.

### 3.4. Failure Load Analysis

**Figure 9:**
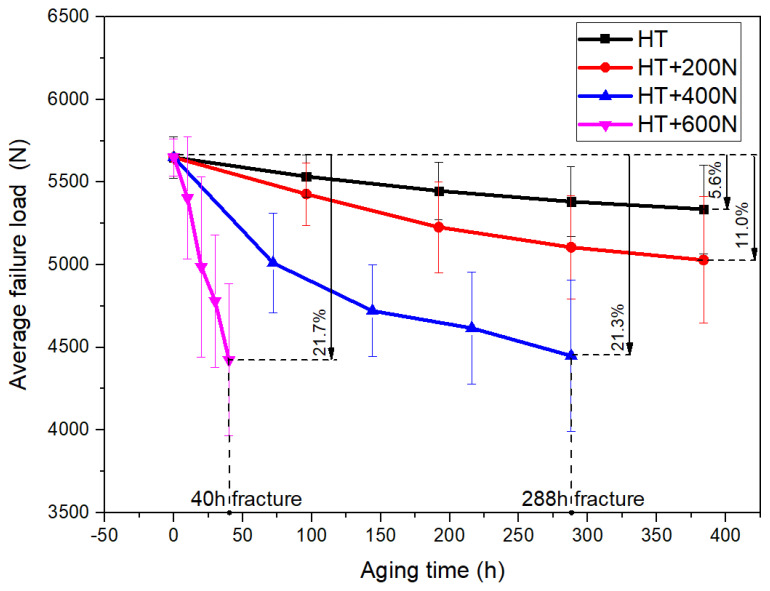
Variation curves of failure load under no-load and loading 200 N, 400 N and 600 N in HT conditions.

**Figure 10:**
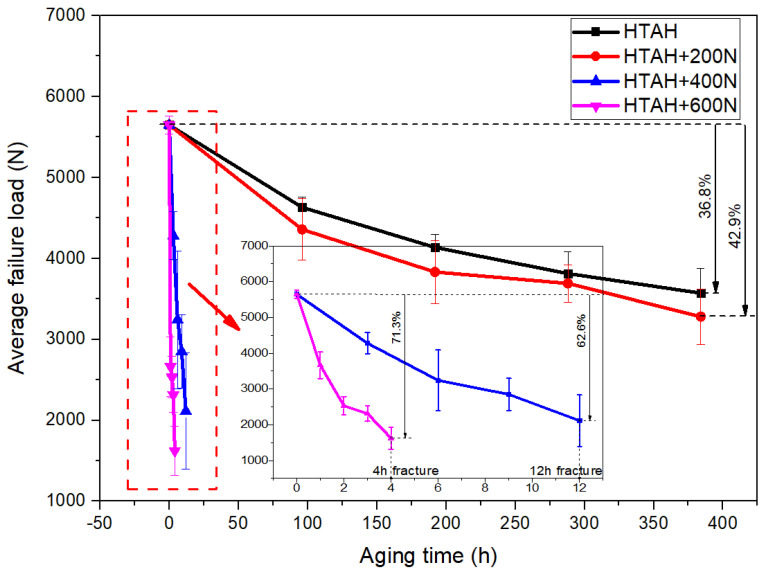
Variation curves of failure load under no-load and loading 200 N, 400 N and 600 N in HTAH conditions.

The coupled environment of temperature, humidity and static load would cause the strength degradation of adhesive joints. Pure aging (no load) and loading 200 N, 400 N and 600 N tests were carried out on joints in HT and HTAH environments, and quasi-static tensile tests were carried out on joints under different environments and load levels to obtain the failure load. Through induction and analysis of the data, the change rule of the failure load with time was obtained, as shown in Figure 9 and Figure 10. Statistical methods were used to analyze the reliability of the experimental data. The mean value and standard deviation were used to calculate the reliability of the experimental data. The experimental data within the 95% confidence interval were extracted, and the average value of the test data was calculated as the representative value of the experimental data.

Compared with nonaging, the failure load decreased by 5.6% after HT aging for 384 h, indicating that HT aging had no obvious effect on the joints. The failure load decreased by 11.0%, and the decrease amplitude increased by 5.4% when loading 200 N at HT. Joint fracture occurred at 240 h with 400 N loading and 40 h with 600 N loading, and the fracture time shortened obviously with increasing load level. Compared with nonaging, the failure load decreased by 21.3% and 21.7% after the maximum cycles of 400 N and 600 N, respectively. When loading was 400 N, the failure load decreased rapidly at first and then slowly, while when loading was 600 N, it decreased almost linearly.

Compared with nonaging, the failure load decreased by 36.8% after HTAH aging for 384 h, and the failure load decreased by 42.9% when loading 200 N at HTAH aging for 384 h, which further decreased by 6.1% compared with no loading. When 400 N and 600 N static loads were applied under HTAH conditions, the failure load decreased significantly. Compared with nonaging, the failure load decreased by 62.6% and 71.3% when loading 400 N and 600 N, respectively, and the failure load decreased almost linearly. Fan et al. [31] studied the hygrothermal aging behavior of the adhesive joint. Through FTIR and DSC chemical analysis of the adhesives before and after aging, they found that hydrolysis led to the fragmentation of the polymer chain, which was the reason for the decline in the adhesive strength after cyclic hygrothermal aging.

In conclusion, under the coupling effect of multiple factors, the failure load of the joint was significantly reduced, and the humidity would cause obvious degradation of the joint’s performance. In high temperature, water molecules accelerated the penetration reaction of adhesive and reduced the intermolecular force of the adhesive. The polymer adhesive underwent easy degradation or cross-linking during the aging process, causing macromolecular chain fracture, leading to reduced bonding performance of the joints [32]. After the joints were loaded, cracks were appeared readily in the adhesive layer, which facilitated the diffusion of water molecules into the adhesive and accelerated the attenuation of joint performance.

### 3.5. Failure Section and Failure Mechanism Analysis

To determine the failure mechanism under the coupling effect of multiple factors, the influence of different environmental factors on the aging failure behavior of polyurethane adhesives could be analyzed by studying the changes in apparent morphology, microstructure and interface during the aging failure process. On the one hand, the failure mode of the joint failure section could be analyzed by analyzing the macromorphology characteristics. On the other hand, SEM micro testing was used to analyze the characteristics of the failure section, such as cracks and holes [33].

**Figure 11:**
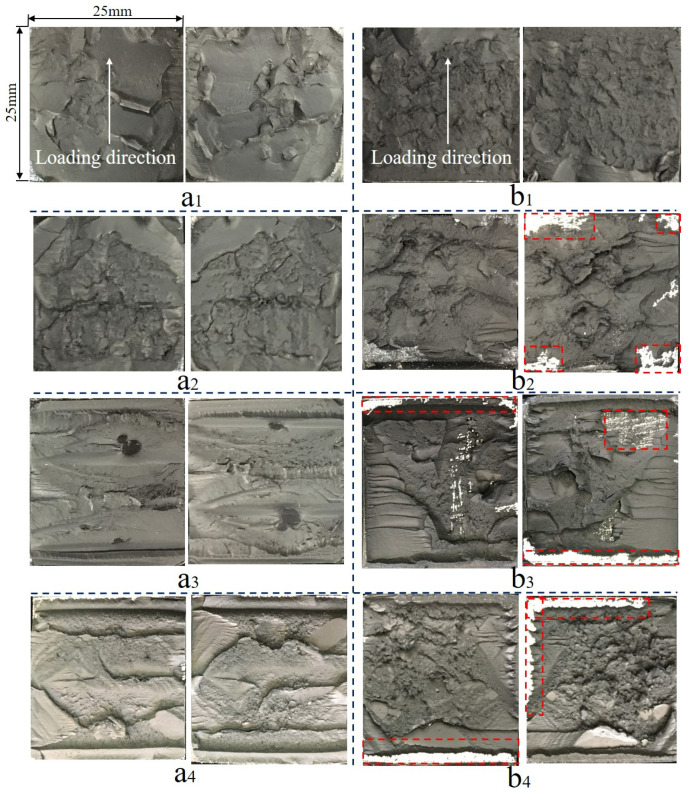
Macrosectional morphology: loading (**a1**) 0 N, (**a2**) 200 N, (**a3**) 400 N and (**a4**) 600 N in the HT environment; loading (**b1**) 0 N, (**b2**) 200 N, (**b3**) 400 N and (**b4**) 600 N in the HTAH environment.

By analyzing the coupling effect of multiple factors on the failure section, typical failure sections under HT and HTAH loading conditions of 0 N (pure aging), 200 N, 400 N and 600 N were obtained, as shown in Figure 11. Arrows indicate the direction of load application. When loading 0 N and 200 N, the failure section was taken at 384 h of aging. Since the joint broke when loading was 400 N and 600 N, the failure sections at the maximum fracture cycle were taken for analysis.

It was found that the failure section mainly showed cohesive failure under HT conditions, and the morphology of the failure section was relatively smooth without loading. With increasing load, the failure section showed an obvious tearing morphology. However, under HTAH conditions, the joint failure section morphology changed significantly, and local interface failure occurred when the load was applied, especially in the edge area. This was because under the effect of load, cracks were more likely to appear at the edge, accelerating the diffusion of water molecules from the boundary of the adhesive layer to the interior. The hydrogen bond interaction between water molecules and polymers led to the expansion of the adhesive [34] and weakening of the binding force.

**Figure 12:**
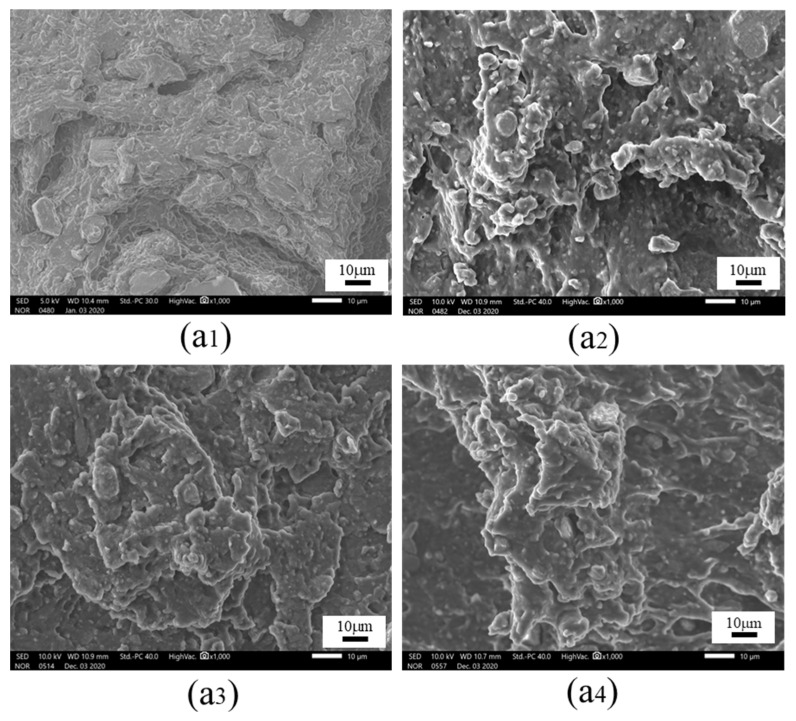
SEM of failure section: loading (**a1**) 0 N, (**a2**) 200 N, (**a3**) 400 N and (**a4**) 600 N under HT environment.

**Figure 13:**
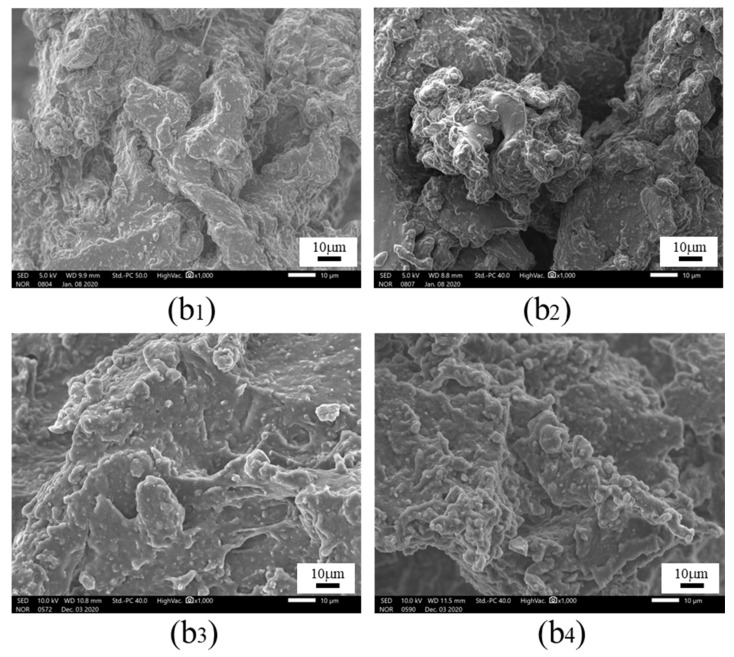
SEM of the failure section: loading (**b1**) 0 N, (**b2**) 200 N, (**b3**) 400 N and (**b4**) 600 N under the HTAH environment.

To further analyze the failure mechanism, scanning electron microscopy (SEM) was selected to conduct high multiple (1000×) analyses on typical failure sections in Figure 11, and the micro sections coupled with 0 N, 200 N, 400 N and 600 N under HT and HTAH conditions were obtained, as shown in Figure 12 and Figure 13. It was found that when 0 N and 200 N were loaded under the condition of HTAH, the molecular structure of the adhesive was changed by water molecules due to the longer aging cycle, degradation of the molecular chain of the adhesive, and tiny particles and microcracks that appeared on the microscopic section. However, when 400 N and 600 N were applied, the joints fractured within a short period, and the microscopic section did not change significantly. The main reason was that the large load could promote the diffusion of water vapor inside the adhesive layer [35], and the volume expansion of the interface adhesive layer occurred, which led to excessive deformation of the structure and failure.

## 4. Conclusions

This paper mainly studied the creep model and degradation behavior under the coupling of multiple factors. The creep deformation was analyzed, and the appropriate creep model was established. Through the temperature, humidity and static load coupling aging test, the change rule of mechanical properties of the joints was obtained. The following conclusions are drawn:

(1) At the same stress level, compared with the HT environment, the creep deformation of the joints at HTAH was more significant, and the creep fracture time of the joints was shorter.

(2) The viscoelastic multiple integral creep model was used to theoretically analyze the creep characteristics of polyurethane adhesive joints. At the same time, the theoretical values of the established creep model were compared with the measured values. It was found that the creep model could better describe the creep behavior of polyurethane bonded joints under uniaxial constant load.

(3) Under the coupling of multiple factors, the failure load of the joints decreased with aging time, and the external load had the most obvious influence on the failure load of the three factors. With the increase of applied load level, the decreasing rate and degree of failure load increased.

(4) During the creep process, the external load made the adhesive layer prone to cracks, facilitating the diffusion of water molecules into the cracks. Water molecules not only easily penetrated into the interface between the adhesive layer/substrate, but also accelerated the degradation of the adhesive.

## Figures and Tables

**Table 1:** Mechanical and physical properties of the test materials.

Material	Young’s Modulus (MPa)	Poisson’s Ratio	Density (kg/m^3^)
Aluminum (6005A)	71,000	0.33	2730
Sikaflex^®^-265	2.7	0.48	1200

**Table 2:** Chemical composition of 6005A aluminum alloy (mass fraction%).

Composition	Fe	Si	Mn	Cr	Cu	Ti	Zn	Mn	Mn + Cr	Other Single	Other Total	Al
Standard	0.35	0.5~0.9	0.5	0.3	0.3	0.1	0.2	0.4~0.7	0.12~0.5	0.05	0.15	Margin

**Table 3:** Testing program.

Environment	Naming of Test Conditions	Static Load/N	Test Period/h
High temperature (80 °C)	HT	0	0, 96, 192, 288, 384
	HT + 200 N	200	
	HT + 400 N	400	Taking the fracture time as the longest cycle, it was equally divided into 5 cycles
	HT + 600 N	600	
High temperature and humidity (80 °C/95% RH)	HTAH	0	0, 96, 192, 288, 384
	HTAH + 200 N	200	
	HTAH + 400 N	400	Taking the fracture time as the longest cycle, it was equally divided into 5 cycles
	HTAH + 600 N	600	

**Table 4:** Characteristic parameters of fitted curves in different environments.

Parameter	Hydrothermal Environment	Stress Level/MPa		
		0.32	0.64	0.96
$\varepsilon_{t}$	HT	0.004	0.0065	0.0034
	HTAH	0.007	0.011	0.024
*n*	HT	0.417	0.374	0.655
	HTAH	0.406	0.968	1.019
$\varepsilon_{0}$	HT	0.02	0.048	0.09
	HTAH	0.032	0.056	0.10

**Table 5:** $\omega_{i}$ values in different environments (i=1~3 ).

Experimental Group	$\omega_{1}$	$\omega_{2}$	$\omega_{3}$
HT	0.0105	0.013	−0.021
HTAH	0.039	−0.0737	0.0615

**Table 6:** $\mu_{i}$ values in different environments (i=1~3 ).

Experimental Group	$\mu_{1}$	$\mu_{2}$	$\mu_{3}$
HT	0.056	0.0095	0.0056
HTAH	0.142	−0.177	0.144

## Data Availability

The data presented in this study are available on request from the corresponding author.
